# The Action and Uses of Pilocarpine

**Published:** 1882-03

**Authors:** 


### THE ACTION AND USES OF PILOCARPINE.

Dr. Squire said that pilocarpine was the alkaloid obtained from the leaves of jaborandi, on which the efficacy of that drug, in producing sweating and salivation, depended. Another alkaloid of different, and even antagonistic properties, named jaborin by Harnack and Meyer, co-existed with it, and rendered the infusion or tincture of jaborandi less certain, and, perhaps, less safe than the pure alkaloid. It was possible that pilocarpin itself had not always been obtained quite free from admixture with jaborin. Muriate of pilocarpin, in simple solution, was the best form to use—one grain to fifteen minims of water for hypodermic injection—and one grain to four ounces of water for internal use, were convenient proportions; one-third grain was the largest, one fifteenth of a grain the smallest dose needed. His plan was to give a full dose at once; others gave small doses every hour with some warm drink or alcoholic stimulant, till perspiration and salivation were freely established. A drachm of the tincture, made with thirty grains of leaf, was equivalent to one-third of a grain of pilocarpin. One-fourth of a grain of the muriate, injected hypodermically, in a few minutes produced suffusion of the face, quickened pulse, some throbbing in the neck, and a general feeling of warmth, followed by free perspiration; this was soon streaming profusely from all parts of the surface, and continued long after the skin had become pale, or even cool; the pulse subsided while the force of the heart's impulse was rather increased; there was a tendency to sleep, and generally a fall of temperature; the perspiration went on for three or four hours; there was an increased flow of saliva, and some increase of pharyngeal, and sometimes of bronchial mucus, that might give rise to bronchial trouble during sleep, and require attention; such a quantity of saliva might be swallowed as to excite vomiting; No headache, sickness, or depression had been noticed by Dr. Squire as a direct result of this medicine.

All the secretions of the body, except the intestinal, were increased by it; the quantity of urine, hardly lessened during the perspiration was increased afterwards. He had not met with dysuria. Swelling and tenderness of the submaxillary salivary glands had remained for a day or two after profuse ptyalism. The action of the drug was on the peripheral secreting apparatus, and not on the nerve-centres, except so far as the first action on the vaso-motor nerves might dilate the vessels, and allow the agent freer access to the glands. Atropia was directly antagonistic to it in this respect. Aconite dilated the vessels, but weakened the heart, while pilocarpin, having no such effect, might even allow small hemorrhage to occur. It was not anæsthetic. The perspiration induced by it did not relieve dysmenorrhœa, sciatica, or colic. Pilocarpin did not moderate specific fevers; but given near the time for the separation of the false membranes in diphtheria, it aided the fall of temperature, and favored sleep. Where there was already a tendency to collapse, of course it could do no good. It was useful in the febrile relapse of scarlatinal nephritis. He had met with no confirmation of the observation that small doses checked perspiration; a similarly homœopathic view, that particular diseases must have particular remedies, had added to the statement that pilocarpin was unsuited to renal disease. The use of it had been chiefly in the different kinds of Bright's disease; it might be unsuited to that particular form where dilated vessels and diminished blood-pressure were associated with a large quantity of albumen; yet, in these cases, it was serviceable to the intercurrent exacerbations and conditions of accidental congestion, not infrequent in their course, and it was preferable to the hot pack, or vapor bath. In the early stages of interstitial nephritis of gouty origin it was of great benefit: in the chronic course these cases generally followed it was often useful; it might be resorted to in some of the extreme effects of renal dropsy, and the relief obtained was not accompanied by great depression. In the chronic results of parenchymatous nephritis, as after scarlet fever, it had been found useful; and that it need not be withheld in some cases of scarlet fever itself was proved by the remarkable results obtained from it by Guttmann in the treatment of diphtheria.—*Trans. Int. Med. Congress.*

